# Analysis of *Kudoa septempunctata* as a cause of foodborne illness and its associated differential diagnosis

**DOI:** 10.4178/epih.e2017037r

**Published:** 2017-08-21

**Authors:** Sung Uk Lee

**Affiliations:** Jeju Special Self-Governing Provincial Office, Jeju, Korea

The difference between South Korea and Japan in terms of animal experiments on the pathogenicity of *Kudoa septempunctata* has not been explored in previous studies. More in-depth discussion among experts is necessary to explain this discrepancy.

Moreover, as you have mentioned, credible case-control studies and retrospective cohort studies have not yet been conducted on *K. septempunctata*. Only animal experiments and case series have been published, which leads me to believe that additional studies are still needed to officially designate *K. septempunctata* as a causative agent of foodborne illness.

As discussed in the paper, the case series study that was presented to argue for a causal link between *K. septempuncatata* and foodborne illness is open to counterargument since the study detected other pathogens as well.

I agree with your opinion that additional epidemiological and pathological investigations are necessary.

